# Knowledge and Attitude Among Lebanese Pregnant Women Toward Cord Blood Stem Cell Storage and Donation

**DOI:** 10.3390/medicina55060244

**Published:** 2019-06-04

**Authors:** Fatima A. Saleh

**Affiliations:** Medical Laboratory Technology Department, Faculty of Health Sciences, Beirut Arab University, Beirut 11-5020, Lebanon; f.saleh@bau.edu.lb

**Keywords:** stem cells, cord blood, pregnant women, knowledge, attitude

## Abstract

*Background*: Umbilical cord blood (UCB) used to be considered waste material and was discarded at birth. However, cord blood is now considered a rich source of adult stem cells that can be used to treat many conditions and diseases. This study was performed to determine pregnant women’s knowledge and attitudes toward cord blood stem cell banking and donation in Lebanon. *Methods*: A descriptive study was conducted in antenatal clinics in Beirut and data were collected using a questionnaire distributed to pregnant women after provision of informed consent. A total of 244 women responded. *Results*: Less than half of the women (46%) reported knowledge about cord blood banking. However, participants with university and secondary education had significantly higher odds of considering UCB storage compared to those with primary education (odds ratio (OR) 8.62, 95% confidence interval (CI) 2.74–27.15 and OR 21.23,95% CI 6.55–68.86, respectively). Older pregnant women were less likely to think about UCB stem cells storage (OR 0.92, 95% CI 0.85–0.98). *Conclusion*: Respondents who had an existing knowledge about UCB stem cells banking in general were more likely to consider storing UCB in blood banks if affordable (45.9%). Therefore, it is necessary to inform pregnant women about stem cell banking so that they can make the appropriate decisions for themselves.

## 1. Introduction

We are always striving to live a long and a healthy life and one of the greatest developments in that direction has come from stem cell research and therapy. Stem cells therapy is a treatment that involves placing stem cells in the part of the body where they are needed to assist in the healing and regeneration of damaged organs. Stem cell theory was first introduced in 1961 by Till and McCulloch who defined stem cells as cells that have the capacity to self-renew and give rise to differentiated progeny [1]. Stem cells, depending on their origin, have been divided into two groups: embryonic stem (ES) cells, which are isolated from the inner cell mass of blastocysts, and adult stem cells, which are found in adult tissues. Due to the unique properties and potential of stem cells, stem cell research has expanded and flourished in an attempt to translate laboratory experiments into clinical applications for the treatment of various genetic and degenerative disorders. Among these are cardiovascular diseases [2], neurodegenerative diseases [3], musculoskeletal diseases [4], autoimmune disorders [5], diabetes [6], cancers [7], as well as many other diseases in the human body that could benefit from stem-cell-based therapies [8,9].

Until recently, umbilical cord blood (UCB) was considered waste material and thus discarded at birth. However, cord blood has been found to be a rich and an invaluable source of stem cells, known as hematopoietic stem cells (HSC), which can be used for the treatment of a variety of diseases such as leukemia, lymphoma, sickle cell disease, and other blood, bone, immune, and metabolic disorders [10,11]. The ease of collection of cord blood stem cells from the newborn’s umbilical cord after birth, coupled with its potential use as a repair kit for health problems that might arise in the future, have led to a steady increase in the number of CB banks [12,13]. Internationally, two kinds of CB banks have been founded: public and private banks. More than 3 million UCB units are stored around the world, with around 650,000 and 2.5 million units in public and private banks, respectively [13]. In Lebanon, only private banks exist; however, no annual data are available on UCB collections that occur.

Most of these banks are agents to international stem cell banks based in Cyprus, the United Kingdom, or Germany (Table S1). These CB banking companies offer collection services in Lebanon; however, the storage of the cord blood units occurs in facilities outside of Lebanon, with costs ranging between $3000 and $4000 per unit for 20 years of storage depending on the company and the product requested [13]. Currently, only two Lebanese companies have opened their facilities for stem cell banking, providing services from collection, processing, storage, and therapy. All these CB banks are located in Beirut and Mount Lebanon Governorates.

Only a small percentage of babies (estimated between 1 in 1000 to 1 in 200,000) ever use the UCB that is stored [14]. The American Academy of Pediatrics 2007 Policy Statement on Cord Blood Banking states that: “Physicians should be aware of the unsubstantiated claims of private cord blood banks made to future parents that promise to insure infants or family members against serious illnesses in the future by use of the stem cells contained in cord blood” [15].

Despite the rise in the number of private banks in Lebanon that sometimes take advantage of limited public awareness, no studies have been conducted so far on a national scale to determine parents’ knowledge and attitude toward storing their child’s umbilical cord blood. This study was conducted for the purpose of determining pregnant women’s knowledge and attitudes toward cord blood stem cell banking and donation in Lebanon.

## 2. Materials and Methods

This study was an exploratory descriptive study that was conducted in antenatal outpatient clinics in Lebanon after approval on February 2016 by the Institutional Review Board of Beirut Arab University with the approval code 2016H-0017-HS-R-123. A total of 244 Lebanese pregnant women above the age of 18 were recruited by convenience sampling from five antenatal outpatient clinics in Beirut during routine prenatal visits. Data were collected using anonymous and self-completed questionnaires. The questionnaires included socio-demographic and obstetric characteristics of participants as well as questions about knowledge and attitudes regarding storing and donating cord blood stem cells. The participants were informed about the aim of the study. Information about anonymity, confidentiality, and consent were included in the explanation, and informed written permission was obtained from each participant. A pilot testing of this questionnaire was conducted to ensure that the wording was appropriate and would yield the required data.

### Data Analysis

Data obtained were analysed using SPSS statistical software version 24 (SPSS, Inc., Chicago, IL, USA. Descriptive statistics for socio-demographic and obstetric characteristics of study participants, as well as their knowledge and attitudes regarding storing and donating cord blood stem cells, are presented as means ± SD and proportions for continuous and categorical variables, respectively. The chi-square (χ2) test or Fisher’s exact test was used to calculate the association between two categorical variables. Independent *t*-tests and Mann–Whitney tests were used to chart comparisons for normal and non-normal continuous variables. Normality of the variables was evaluated using the Kolmogorov–Smirnov and Shapiro–Wilk tests of normality. The association between socio-demographic and obstetric characteristics of pregnant women and their attitudes regarding storage of UCB stem cells was assessed using one-way analysis of variance (ANOVA) and Chi-square tests. The associations between socio-demographic and obstetric characteristics of pregnant women and their attitudes regarding storage of UCB stem cells were explored using multinomial logistic regression, with participants’ attitude as the outcome variable. The associations of socio-demographic and obstetric characteristics of pregnant women and their knowledge regarding storage of UCB stem cells, as well as attitudes regarding donation of cord stem cell for research purposes, were also assessed using simple and multiple logistic models. Odds ratios (OR) and their respective 95% confidence intervals (CIs) were computed. Statistical significance was detected by a *p*-value < 0.05.

## 3. Results

### 3.1. Participant Characteristics

The survey was conducted over six months from July 2017 to February 2018, in which 244 pregnant women completed the survey about UCB stem cell banking. The mean age of the pregnant women was 28.81 ± 6.61 years and 73.7% were city residents. Most of the women (70.8%) in the study population were in their first or second trimester and had at least one child (64.7%). Regarding educational status, 51.6% had graduate level education, 28.3% had completed their secondary education, and 20.1% had finished primary education, as shown in Table 1. Around three-quarters of women (74.7%) had a family monthly income equal or below $2000.

### 3.2. Knowledge and Attitudse of Participants Toward UCB Stem Cells Banking

Less than half of the respondents (46.1%) knew about UCB stem cell banking. Only 56.4% received the information from their physicians, whereas, 20.8% through media, and 22.8% via the Internet. More than half the respondents (66.4%) who knew about cord blood banking were aware of its storage costs, as shown in Table 2. Regarding participants’ attitudes toward UCB stem cell storage, 28.7% refused, 23% would think about it, 37.7% showed willingness for UCB stem cell storage if it was affordable, and only 10.7% agreed to store UCB stem cells if it was used for their own family members. Half the respondents (51.6%) would donate UCB stem cells for research purposes and a smaller proportion (41%) of participants would donate UCB stem cells to treat patients outside family, as shown in Table 2.

Table 3 provides the simple and multiple logistic regression analysis used to explore the association of socio-economic and obstetric characteristics with knowledge toward UCB stem cell banking among the study population. Using simple logistic regression, factors significantly associated with knowledge regarding UCB stem cell banking included age, educational level, family income, residency status, stage of pregnancy, and number of children. In particular, participants who belonged to the secondary education group or those with university education had significantly higher odds of knowing about UCB stem cells banking compared to the primary school participants (OR: 2.52, 95% CI (1.05, 6.05)) and (OR: 6.95, 95% CI (3.09, 15.62), respectively). Participants with middle and high income levels were approximately 3.89 and 6.35 times more likely to know about UCB stem cells banking compared with those with lower income levels (OR: 3.89 95% CI (2.02, 7.46)), respectively. Participants living in the city were 99% more knowledgeable about UCB stem cells banking than those who did not. Finally, having one previous child increased women’s chance for a better knowledge regarding UCB stem cells banking compared to those who had no previous children (OR 3.42, 95% CI (1.78, 6.56))

A multivariate logistic regression model (Table 3) was created to explore the association of socio-economic and obstetric characteristics with knowledge toward UCB stem cell banking among the study population. Variables were input to the model in order of strength of their association with the knowledge regarding UCB stem cell banking as per the simple logistic analysis. The variables included in the final adjusted model were based on the degree of importance or relavance of this indpendnet variable with the main outcome. The final model included the following variables: age, educational level, family income, residency status, number of children, and stage of pregnancy. The results of the multiple analysis showed that educational level, family income, residency status, and number of children were significantly associated with knowledge regarding UCB stem cell banking. Respondents with a university education were 5.1 times more likely to know about UCB stem cells banking compared to those with primary education (OR 5.13, 95% CI 2.05–12.85). Participants with middle and high income levels were approximately 2.8 and 4.6 times more likely to know about UCB stem cells banking compared with those with a lower income (OR 2.85, 95% CI 1.32–6.61 and OR 4.56, 95% CI 1.81–11.53, respectively). Having one previous child and living in city increased women’s chance for better knowledge of UCB stem cells banking compared to those who had no previous children (OR 4.27, 95% CI 1.86–9.81 and OR 2.18, 95% CI 1.06–4.49, respectively).

Table 4 presents the association between socio-economic and obstetric characteristics of pregnant women and their attitude regarding storage of UCB stem cells. As shown in Table 4, participants who refused to store UCB stem cells had a significantly higher mean age compared to those who would think about storage (30.3 ± 7.2 years vs. 26.9 ± 6.6 years, *p* = 0.021). Of participants who agreed to store UCB stem cells for family members, 65.4% had a university education. Similarly, 46.2% of participants who agreed to store UCB stem cells for family members had the highest monthly income level (>$2000). The vast majority of city residents had a positive attitude toward UCB stem cells banking compared to non-city residents (82.1% will think about it, 81.3% will do it if affordable, 73.1% will do if used for family, *p* = 0.002).

Multinomial logistic regression analyses (Table 5) were used to explore the association of socio-economic and obstetric characteristics with attitude toward UCB stem cell banking among the study population. Age, educational level, family income, and residency status remained significantly associated with women’s attitudes toward UCB stem cells storage. Respondents who were city residents were more likely to consider UCB stem cells storage if affordable (OR 2.92, 95% CI 1.31–6.51) and if they thought about UCB stem cells storage twice (OR 3.03, 95% CI 1.25–7.34). Participants with university and secondary education had significantly higher odds of considering UCB stem cells storage if affordable compared to those with primary education (OR 8.62, 95% CI 2.74–27.15 and OR 21.23, 95% CI 6.55–68.86, respectively). Women in the highest family income level were less likely to consider UCB stem cells storage if affordable (OR 0.31, 95% CI 0.11–0.93). Older pregnant women were less likely to think about UCB stem cells storage (OR 0.92, 95% CI 0.85–0.98) (Table 5).

### 3.3. Donation to Research

Table 6 provides the simple and multiple logistic regression analysis used to explore the association of socio-economic and obstetric characteristics of pregnant women and their attitudes regarding donation of cord stem cell for research purposes among the study population. Using simple and multiple logistic regression, factors significantly associated with attitude regarding donation of cord stem cells for research purposes included age, educational level, and residency status. Using multivariate logistic regression analysis (Table 6), respondents with secondary and university education were 3.20 and 4.45 times more likely, respectively, to show willingness to donate UCB stem cells for research purposes compared to those with primary education (OR 3.20, 95% CI 1.39–7.34 and OR 4.45, 95% CI 1.99–9.98, respectively). Compared with non-city residents, respondents living in a city had greater odds of donating UCB stem cells for research purposes (OR 2.06, 95% CI 1.10–3.87). Respondents with a university education were 2.5 times more likely to show willingness to donate UCB stem cells to treat patients outside their family compared to those with primary education (OR 2.52, 95% CI 1.322–5.19) (Supplementary Materials).

## 4. Discussion

Lebanon is an important market for cord blood stem cell banking because of its high birth rate. Consequently, private banks are on the rise, but storage is expensive and controversy has been surrounding them, especially due to the absence of clear regulations to govern the status of stem cell banks. The slow development of public CB banks in Lebanon is likely due to the political and economic instabilities in the country, as public banks are primarily funded by governments or charitable resources [13]. However, before a public bank can operate in Lebanon, regulations and policies to govern public CB banks are urgently needed so that we do not lag far behind the rest of the Arab world, where significant progress has been made in this field.

The current widespread public discussion on cord blood stem cells and the many related issues involving has highlighted the need to determine pregnant women’s knowledge and attitudes toward this topic.

In our study, when pregnant women were asked about their knowledge about UCB stem cell banking, over half of the participants (54%) reported no prior knowledge. Similar to the study by Dinc and Sahin [16] which examined knowledge of Turkish participants about cord blood banking, found that around 70% had poor or very poor knowledge, which was similar to the findings reported by of Fernandez et al. [17]. This lack of knowledge is consistent with other studies, such as the one conducted by Jawdat et al. [18], in which more than half of the Saudi Arabian respondents were unaware of CB banking, and the study by Matsumto et al. [19] showing that 75% of women participants knew nothing about CB banking in Jordan. However, better awareness was indicated in Australian pregnant women, where 70% of the respondents had adequate knowledge about CB banking [20,21].

More than half of the participants (56.4%) who had knowledge reported their physician as the primary source of information. This was surprising especially as another study conducted on women from five European countries found that only 20.6% received information from a healthcare provider, whereas the majority obtained information through mass media [22]. This finding echoes other studies showing that the main source of information for pregnant women was media, whereas less than 10% obtained information from medical personnel [19,23].

Here, we highlight the importance of obtaining scientific information from the physician rather than potentially misleading or inaccurate information through media. Women with higher educational levels had more knowledge about UCB banking, which was in accordance with other studies [16,17]. Middle- and high-income participants with one previous child and living in the city had a greater chance of better UCB knowledge. Similar results were reported in a more recent study conducted in Jordan, where women of higher socio-economic status reported a higher level of knowledge about CB banking [24].

As expected, respondents who had existing knowledge about UCB stem cell banking had a more positive attitude toward UCB donation, with 64% of subjects willing to donate for research purposes and 51.4% for treating patients outside family members. Katz et al. reported that almost 92% of women who wished to store their child’s cord blood would be willing to donate it to research when it is not suitable for transplantation [22]. However, this study was conducted in Europe, whereas in Lebanon, there are some cultural misconceptions regarding scientific research in general, and for many individuals, tissue donation continues to be considered taboo. In Lebanon, the majority of parents are donating their baby’s cord blood for their personal and family’s potential future use. Like other Arabic cultures, Lebanese people are taught to make donations of their money, property, or time, but not of themselves [25]. Moreover, there is no clear regulation in Lebanon to govern the status of stem cell banks. Therefore, considerable education efforts about the subject are required to increase public awareness and to work toward adopting a regulatory law that controls stem cell use in Lebanon [26]. These measures would undoubtedly increase public awareness and subsequently the market size for stem cell banking.

Despite its importance, some limitations should be considered when interpreting the study findings. First, the majority of participants in the study were educated at the secondary or university-educational level. Second, the study was conducted in antenatal outpatient clinics in Beirut, which is the capital of Lebanon. This would have a potential limitation to the generalizability of the study findings. Ideally, a random sample should have been obtained from other socio-geographic areas of the country, which, together with Beirut, would represent a national representative sample of the Lebanese population. Another limitation lies in the size of the sample; however, due to lack of resources in the setting of the study, it was not possible to obtain a larger sample size. Among the factors thought to affect attitude toward cord blood stem cell banking is religion. However, given the political tension in the country, no data about religious beliefs were collected.

## 5. Conclusions

In conclusion, pregnant women who had previous knowledge about UCB stem cells banking were more likely to consider storing UCB in blood banks if affordable. Therefore, it is necessary to inform pregnant women about stem cell banking so that they can make the decision that is right for themselves. To the best of our knowledge, this study is the first to provide evidence on cord blood stem cell banking, which is still in its infancy in Lebanon, and such studies are needed as the data can be used in the development of appropriate policies and procedures for cord blood stem cell banking.

## Figures and Tables

**Table 1:** Socio-demographic and obstetric characteristics of pregnant women responding to a survey about umbilical cord blood (UCB) stem cells banking (*n* = 244).

Variable	Total Sample (*n* = 244)
Age, Mean ± SD	28.81 ± 6.61
Educational level, *n* (%)	
Primary school	49 (20.1)
Secondary school	69 (28.3)
University	126 (51.6)
Family monthly income, *n* (%)	
<$1000	86 (35.7)
$1000–$2000	94 (39.0)
>$2000	61 (25.3)
City resident, *n* (%)	
No	64 (26.3)
Yes	179 (73.7)
Stage of pregnancy, *n* (%)	
First trimester	90 (37.7)
Second trimester	79 (33.1)
Third trimester	70 (29.3)
Number of children, *n* (%)	
0	86 (35.2)
1	76 (31.1)
≥2	82 (33.6)

**Table 2:** Knowledge, attitudes, and perceptions of pregnant women toward UCB stem cells banking (*n* = 244).

Knowledge & Attitude	Total Sample (*n* = 244)
Knowledge of UCB stem cells banking, n (%)	
No	130 (53.9)
Yes	111 (46.1)
Source of information (*n* = 101), *n* (%)	
Doctor	57 (56.4)
Media	21 (20.8)
Internet	23 (22.8)
Awareness of cost of UCB stem cells storage (*n* = 116), *n* (%)	
No	39 (33.6)
Yes	77 (66.4)
Would like to receive information about UCB stem cells banking, *n* (%)	
No	67 (27.7)
Yes	175 (72.3)
Would store in a UCB stem cells blank, *n* (%)	
Will never do	70 (28.7)
Will think about it	56 (23.0)
Will definitely do if it is affordable	92 (37.7)
Will definitely do if it is only used for my own family	26 (10.7)
Would donate UCB stem cells for research purposes, *n* (%)	
No	118 (48.4)
Yes	126 (51.6)
Would donate UCB stem cells to be used to treat patients outside family, *n* (%)	
No	144 (59.0)
Yes	100 (41.0)

**Table 3:** Multivariate logistic regression analysis of socio-economic and obstetric characteristics of pregnant women and their knowledge regarding storage of UCB stem cells (*n* = 244).

	Knowledge Regarding Cord Blood Banking				
	No	Yes	Statistical Significance	Unadjusted OR (95% CI)	Adjusted OR (95% CI)
Age, Mean ± SD	27.8 ± 6.9	30.2 ± 5.9	*p* = 0.004	1.06 (1.02,1.10)	1.03 (0.98,1.09)
Educational level, *n* (%)			*p* < 0.001		
Primary school	39 (30.0)	9 (8.1)		1.00	1.00
Secondary school	43 (33.1)	25 (22.5)		2.52 (1.05,6.05)	1.53 (0.58,4.03)
University	48 (36.9)	77 (69.4)		6.95 (3.09,15.62)	5.13 (2.05,12.85)
Family monthly income, *n* (%)			*p* < 0.001		
<$1000	65 (50.0)	19 (17.6)		1.00	1.00
$1000–2000	44 (33.8)	50 (46.3)		3.89 (2.02,7.46)	2.85 (1.32,6.61)
>$2000	21 (16.2)	39 (36.1)		6.35 (3.04,13.27)	4.56 (1.81,11.53)
City resident, *n* (%)			*p* = 0.023		
No	41 (31.8)	21 (18.9)		1.00	1.00
Yes	88 (68.2)	90 (81.1)		1.997 (1.09,3.65)	2.18 (1.06,4.49)
Stage of pregnancy, *n* (%)			NS		
First trimester	48 (37.5)	42 (38.9)		1.00	1.00
Second trimester	46 (35.9)	32 (29.6)		0.795 (0.43,1.47)	−0.37 (0.17,0.83)
Third trimester	34 (26.6)	34 (31.5)		1.14 (0.61,2.15)	0.35 (0.14,0.84)
Number of children, *n* (%)			*p* = 0.001		
0	57 (43.8)	27 (24.3)		1.00	1.00
1	29 (22.3)	47 (42.3)		3.42 (1.78,6.56)	4.27 (1.86,9.81)
≥2	44 (33.8)	37 (33.3)		1.77 (0.94,3.34)	2.03 (0.85,4.88)

**Table 4:** Association between socio-economic and obstetric characteristics of pregnant women and their attitude regarding storage of UCB stem cells. NS denotes not significant.

Variable	Attitude Regarding Storage of Cord Stem Cells				
	Never	Will Think About It	Will Do If Affordable	Will Do If Used for Family	Significance
Age, Mean ± SD	30.3 ± 7.2	26.9 ± 6.6	28.3 ± 5.9	30.9 ± 6.4	*p* = 0.021
Educational level, *n* (%)					*p* < 0.001
Primary school	25 (35.7)	14 (25.0)	6 (6.5)	4 (15.4)	
Secondary school	22 (31.4)	14 (25.0)	28 (30.4)	5 (19.2)	
University	23 (32.9)	28 (50.0)	58 (63.0)	17 (65.4)	
Family monthly income, *n* (%)					*p* = 0.024
<$1000	27 (39.1)	15 (26.8)	40 (44.4)	4 (15.4)	
$1000–2000	24 (34.8)	25 (44.6)	35 (38.9)	10 (38.5)	
>$2000	18 (26.1)	16 (28.6)	15 (16.7)	12 (46.2)	
City resident, *n* (%)					*p* = 0.002
No	30 (42.9)	10 (17.9)	17 (18.7)	7 (26.9)	
Yes	40 (57.1)	46 (82.1)	74 (81.3)	19 (73.1)	
Stage of pregnancy, *n* (%)					NS
First trimester	25 (36.2)	19(34.5)	39 (42.9)	7 (29.2)	
Second trimester	20 (29.0)	25 (45.5)	26 (28.6)	8 (33.3)	
Third trimester	24 (34.8)	11 (20.0)	26 (28.6)	9 (37.5)	
Number of children, *n* (%)					NS
0	19 (27.1)	20 (35.7)	39 (42.4)	8 (30.8)	
1	23 (32.9)	22 (39.3)	22 (23.9)	9 (34.6)	
≥2	28 (40.0)	14 (25.0)	31 (33.7)	9 (34.6)	

**Table 5:** Multinomial logistic regression analysis between socio-economic and obstetric characteristics of pregnant women and their attitudes regarding storage of UCB stem cells.

	Unadjusted OR (95% CI)			Adjusted OR (95% CI)		
	Will Think About It	Will Do if Affordable	Will Do if Used for Family	Will Think About It	Will Do if Affordable	Will Do if Used for Family
Age, Mean ± SD	0.92 (0.87,0.97)	0.95 (0.91,1.00)	1.01 (0.95,1.09)	0.92 (0.85,0.98)	0.976 (0.91,1.031)	1.00 (0.923,1.098)
Educational level, *n* (%)						
Primary school	1.00	1.00	1.00	1.00	1.00	1.00
Secondary school	1.14 (0.44,2.90)	5.30 (1.85,15.18)	1.42 (0.34,5.96)	1.42 (0.47,4.23)	8.62 (2.74,27.15)	0.86 (0.17,4.39)
University	2.17 (0.92,5.11)	10.51 (3.81,28.95)	4.62 (1.35,15.77)	2.98 (1.01,8.79)	21.23 (6.55,68.86)	3.19 (0.75,13.48)
Family monthly income, *n* (%)						
<$1000	1.00	1.00	1.00	1.00	1.00	1.00
$1000–2000	1.87 (0.81,3.36)	0.98 (0.48,2.01)	2.81 (0.78,10.15)	1.90 (0.71,5.13)	0.65 (0.26,1.59)	2.98 (0.66,13.52)
>$2000	1.60 (0.64,4.03)	0.56 (0.24,1.30)	4.50 (1.25,16.17)	1.95 (0.58,6.52)	0.31 (0.11,0.93)	3.66 (0.71,18.88)
City resident, *n* (%)						
No	1.00	1.00	1.00	1.00	1.00	1.00
Yes	3.45 (1.50,7.92)	3.26 (1.61,6.63)	2.04 (0.76,5.46)	3.03 (1.25,7.34)	2.92 (1.31,6.51)	1.61 (0.56,4.61)
Stage of pregnancy, *n* (%)						
First trimester	1.00	1.00	1.00	1.00	1.00	1.00
Second trimester	1.64 (0.71,3.80)	0.83 (0.39,1.80)	1.43 (0.44,6.61)	1.83 (0.68,4.90)	1.24 (0.48,3.19)	1.65 (0.44,6.158)
Third trimester	0.60 (0.24,1.53)	0.69 (0.33,1.47)	1.34 (0.43,4.17)	0.57 (0.19,1.75)	0.68 (0.26,1.75)	1.08 (0.27,4.28)
Number of children, *n* (%)						
0	1.00	1.00	1.00	1.00	1.00	1.00
1	0.91 (0.38,2.14)	0.47 (0.21,1.04)	0.93 (0.30,2.88)	0.81 (0.29,2.27)	0.60 (0.23,1.57)	1.32 (0.36,4.82)
≥2	0.47 (0.19,1.17)	0.54 (0.25,1.14)	0.76 (0.25,2.33)	1.11 (0.35,3.50)	1.48 (0.53,4.10)	2.53 (0.60,1.075)

**Table 6:** Multivariate logistic regression analysis of socio-economic and obstetric characteristics of pregnant women and their attitudes regarding donation of cord stem cell for research purposes. NS denotes not significant.

Variable	Attitude Toward Cord Blood Banking Donation for Research Purposes				
	No	Yes	Statistical Significance	Unadjusted OR (95% CI)	Adjusted OR (95% CI)
Age, Mean ± SD	29.69 ± 7.17	27.98 ±5.94	*p* = 0.044	0.961 (0.92,0.99)	0.97 (0.93,1.02)
Educational level, *n* (%)			*p* = 0.01		
Primary school	33 (28.0)	16 (12.7)		1.00	1.00
Secondary school	32 (27.1)	37 (29.4)		2.38 (1.11,5.11)	3.20 (1.39,7.34)
University	53 (44.9)	73 (57.9)		2.84 (1.42,5.69)	4.45 (1.99,9.98)
Family monthly income, *n* (%)			NS		
<$1000	42 (36.2)	44 (35.2)		1.00	1.00
$1000–2000	40 (34.5)	54 (43.2)		1.29 (0.72,2.32)	1.09 (0.54,2.17)
>$2000	34 (29.3)	27 (21.6)		0.76 (0.39,1.46)	0.64 (0.28,1.47)
City resident, *n* (%)			*p* = 0.004		
No	41 (34.7)	23 (18.4)		1.00	1.00
Yes	77 (65.3)	102 (81.6)		2.36 (1.31,4.26)	2.06 (1.10,3.87)
Stage of pregnancy, *n* (%)			NS		
First trimester	43 (37.7)	47 (37.6)		1.00	1.00
Second trimester	31 (27.2)	48 (38.4)		1.42 (0.77,2.61)	1.76 (0.86,3.57)
Third trimester	40 (35.1)	30 (24.0)		0.69 (0.37,1.29)	0.72 (0.34,1.52)
Number of children, *n* (%)			NS		
0	37 (31.4)	49 (38.9)		1.00	1.00
1	38 (32.2)	38 (30.2)		0.755 (0.41,1.40)	0.67 (0.32,1.39)
≥2	43 (36.4)	39 (31.0)		0.68 (0.37,1.26)	0.93 (0.42,2.06)

## Supplementary Materials

The following are available online at https://www.mdpi.com/1010-660X/55/6/244/s1. Table S1: Available cord blood (CB) banks in Lebanon.
