# Supplementary material for: Knowledge and Attitude Among Lebanese Pregnant Women Toward Cord Blood Stem Cell Storage and Donation
Source: Medicina (Kaunas). 2019 Jun 4;55(6):244. doi: 10.3390/medicina55060244 (PMC6630680; doi:10.3390/medicina55060244)
Supplement: Supplementary file 1 [file medicina-55-00244-s001.pdf]

**Table S1.** Available cord blood (CB) banks in Lebanon.

| CB bank | Storage Lab | Accreditation |
|---|---|---|
| Biomax | Leipzig, Germany | FACT |
| Biovault | Plymouth, UK | AABB, HTA, ISO, JACIE |
| Cells4Life | Essex, UK | HTA, ISO |
| Future Health | Nottingam, UK | AABB, HTA, ISO |
| Lifeline Services | Nicosia, Cyprus | AABB, ISO |
| Reviva | Metn, Lebanon | |
| Smart cells | West Drayton, UK | HTA, ISO |
| Transmedical For Life | Beirut, Lebanon | |
